# Supplementary material for: The eGFR Decline as a Risk Factor for Metabolic Syndrome in the Korean General Population: A Longitudinal Study of Individuals with Normal or Mildly Reduced Kidney Function
Source: Biomedicines. 2023 Apr 5;11(4):1102. doi: 10.3390/biomedicines11041102 (PMC10135887; doi:10.3390/biomedicines11041102)
Supplement: Supplementary file 1 [file biomedicines-11-01102-s001.zip › biomedicines-2243690-supplementary.pdf]

Supplementary Table S1. Baseline demographics, clinical characteristics of total study participants based on by eGFR category (*n* = 7107)

**Characteristics**	**eGFR category, ml/min/1.73 m^2**					***p* value**
	**Total (*n* = 7107)**	**eGFR ≥ 105 (*n* = 2114)**	**eGFR 90 to < 105 (*n* = 3872)**	**eGFR 75 to < 90 (*n* = 956)**	**eGFR 60 to < 75 (*n* = 165)**	
**Demographic and clinical data**						
Age (year)	55.8 ± 8.7	48.5 ± 4.6	57.6 ± 7.9	62.6 ± 8.4	64.5 ± 7.4	< 0.001
Male (%)	3387 (47.7%)	921 (43.6%)	1899 (49.0%)	487 (50.9%)	80 (48.5%)	< 0.001
BMI (kg/m^2)	24.5 ± 3.1	24.2 ± 3.0	24.5 ± 3.1	24.7 ± 3.2	25.2 ± 3.7	< 0.001
Exercise (MET/d)	120.7 ± 237.4	119.2 ± 270.0	119.4 ± 219.8	127.8 ± 224.3	127.8 ± 261.3	0.558
Alcohol consumption (g/day)	9.1 ± 20.8	10.8 ± 23.6	8.9 ± 19.8	7.2 ± 17.9	5.9 ± 15.1	0.001
Waist circumference (cm)	84.4 ± 8.8	82.0 ± 8.6	85.1 ± 8.7	86.5 ± 8.5	87.8 ± 10.2	< 0.001
Smoking status						0.835
Never smoker	4443 (62.5%)	1368 (64.7%)	2401 (62.0%)	579 (60.6%)	95 (57.6%)	
Ex-smoker	1294 (18.2%)	304 (14.4%)	727 (18.8%)	220 (23.0%)	43 (26.1%)	
Current smoker	1370 (19.3%)	442 (20.9%)	744 (19.2%)	157 (16.4%)	27 (16.4%)	
Education						< 0.001
< 6 years	2242 (31.5%)	328 (15.5%)	1444 (37.3%)	390 (40.8%)	80 (48.5%)	
6 to < 12 years	3822 (53.8%)	1419 (67.1%)	1926 (49.7%)	418 (43.7%)	59 (35.8%)	
≥ 12 years	1043 (14.7%)	367 (17.4%)	502 (13.0%)	148 (15.5%)	26 (15.8%)	
eGFR (ml/min/1.73 m^2)	99.2 ± 9.9	109.6 ± 4.2	98.3 ± 4.2	85.0 ± 4.0	69.5 ± 4.3	< 0.001
Fasting glucose (mg/dL)	92.2 ± 14.5	91.0 ± 14.0	92.4 ± 14.5	93.8 ± 15.6	92.6 ± 13.2	0.121
SBP (mmHg)	116.4 ± 16.4	111.5 ± 14.5	117.8 ± 16.6	120.6 ± 17.0	122.5 ± 17.6	< 0.001
DBP (mmHg)	77.7 ± 10.2	76.2 ± 10.4	78.4 ± 10.1	78.5 ± 10.1	77.7 ± 9.8	0.059
HDL (mg/dL)	44.1 ± 10.2	45.6 ± 10.8	43.9 ± 10.1	42.3 ± 9.4	40.5 ± 9.4	< 0.001
TG (mg/dL)	141.4 ± 106.9	133.1 ± 117.6	141.4 ± 100.7	155.1 ± 107.8	166.8 ± 86.4	< 0.001
HTN (%)	2174 (30.6%)	387 (18.3%)	1247 (32.2%)	441 (46.1%)	99 (60.0%)	< 0.001
DM (%)	1177 (16.6%)	243 (11.5%)	620 (16.0%)	250 (26.2%)	64 (38.8%)	< 0.001
Metabolic syndrome (%)	2295 (32.3%)	453 (21.4%)	1303 (33.7%)	441 (46.1%)	98 (59.4%)	< 0.001
**MS components**						
Abdominal obesity (%)	3319 (46.7%)	785 (37.1%)	1907 (49.3%)	534 (55.9%)	93 (56.4%)	< 0.001
High blood pressure (%)	3157 (44.4%)	637 (30.1%)	1811 (46.8%)	583 (61.0%)	126 (76.4%)	< 0.001
Hypertriglyceridemia (%)	2238 (31.5%)	558 (26.4%)	1218 (31.5%)	384 (40.2%)	78 (47.3%)	< 0.001
High fasting glucose (%)	956 (13.5%)	201 (9.5%)	491 (12.7%)	216 (22.6%)	48 (29.1%)	< 0.001
Low HDL (%)	4008 (56.4%)	1121 (53.0%)	2171 (56.1%)	601 (62.9%)	115 (69.7%)	< 0.001

Data are presented as mean ± standard deviation. BMI, body mass index; eGFR, estimated glomerular filtration rate; SBP, systolic blood pressure; DBP, diastolic blood pressure; HDL, high density lipoprotein; TG, triglyceride; MET, metabolic equivalent of task

Supplementary Table S2. Association of prevalence MS based on eGFR category at baseline (n = 7107)

**eGFR category, ml/min/1.73 m²**		**Odds ratio (95% CI) *p* value**				***p for* linear trend**
		eGFR ≥ 105	eGFR 90 to < 105	eGFR 75 to < 90	eGFR 60 to < 75	
**Total**	**Prevalence n, (%)**	453 (21.4%)	1303 (33.7%)	441 (46.1%)	98 (59.4%)	
	Crude	Reference	1.860 (1.644–2.104)<br>< 0.001	3.140 (2.664–3.700)<br>< 0.001	5.363 (3.865–7.442)<br>< 0.001	< 0.001
	Model 1	Reference	1.332 (1.152–1.540)<br>< 0.001	2.073 (1.712–2.511)<br>< 0.001	3.379 (2.381–4.796)<br>< 0.001	< 0.001
	Model 2	Reference	1.338 (1.156–1.549)<br>< 0.001	2.141 (1.764–2.599)<br>< 0.001	3.446 (2.42–4.906)<br>< 0.001	< 0.001
	Model 3	Reference	1.23 (1.054–1.435)| 0.008	1.921 (1.563–2.361)<br>< 0.001	3.156 (2.168–4.594)<br>< 0.001	< 0.001
**Women**	Prevalence n, (%)	255 (21.4%)	836 (42.4%)	282 (60.1%)	62 (72.9%)	
	Crude	Reference	2.705 (2.294–3.189)<br>< 0.001	5.547 (4.403–6.988)<br>< 0.001	9.916 (6.025–16.318)<br>< 0.001	< 0.001
	Model 1	Reference	1.313 (1.071–1.609)<br>0.009	2.280 (1.739–2.990)<br>< 0.001	3.415 (2.016–5.786)<br>< 0.001	< 0.001
	Model 2	Reference	1.307 (1.064–1.606)<br>0.011	2.308 (1.754–3.036)<br>< 0.001	3.407 (2.005–5.788)<br>< 0.001	< 0.001
	Model 3	Reference	1.286 (1.037–1.594)<br>0.022	2.213 (1.661–2.949)<br>< 0.001	3.317 (1.911–5.756)<br>< 0.001	< 0.001
**Men**	Prevalence n, (%)	198 (21.5%)	467 (24.6%)	159 (32.6%)	36 (45.0%)	
	Crude	Reference	1.191 (0.986–1.438)<br>0.070	1.770 (1.384–2.264)<br>< 0.001	2.989 (1.873–4.772)<br>< 0.001	< 0.001
	Model 1	Reference	1.113 (0.906–1.367)<br>0.310	1.679 (1.276–2.208)<br>< 0.001	2.813 (1.734–4.562)<br>< 0.001	< 0.001
	Model 2	Reference	1.124 (0.913–1.384)<br>0.270	1.707 (1.291–2.257)<br>0.000	2.854 (1.751–4.654)<br>< 0.001	< 0.001
	Model 3	Reference	0.984 (0.788–1.23)<br>0.887	1.464 (1.081–1.982)<br>0.014	2.598 (1.523–4.429)<br>0.001	0.001

Data are presented as Odds ratio (95% Confidential incidence) and *p*-value; Model 1: adjusted for age and sex; Model 2: adjusted for age, sex, education, smoking, exercise, and alcohol consumption; Model 3: adjusted for age, sex, education, smoking, exercise, alcohol consumption, and BMI
